# Saying and doing are different things: a scoping review on how health equity is conceptualized when considering healthcare system performance

**DOI:** 10.1186/s12939-023-01872-z

**Published:** 2023-07-13

**Authors:** Nakia K. Lee-Foon, Victoria Haldane, Adalsteinn Brown

**Affiliations:** 1 Dalla Lana School of Public Health, University of Toronto, 155 College Street, 6th Floor, Toronto, ON M5T 3M7 Canada; 2 Institute of Health Policy, Management and Evaluation, University of Toronto, 155 College Street, 4th Floor, Toronto, ON M5T 3M6 Canada

**Keywords:** Equity, Equality, Disparities, Social determinants of health, Healthcare, Health inequities, Healthcare systems, Healthcare institutions

## Abstract

**Introduction:**

Ensuring healthcare systems provide equitable, high quality care is critical to their users’ overall health and wellbeing. Typically, systems use various performance frameworks and related indicators to monitor and improve healthcare. Although these frameworks usually include equity, the extent that equity is reflected in these measurements remains unclear. In order to create a system that meets patients’ needs, addressing this uncertainty is important. This paper presents findings from a scoping review that sought to answer the question ‘How is equity conceptualized in healthcare systems when assessing healthcare system performance?’.

**Methods:**

Levac’s scoping review approach was used to locate relevant articles and create a protocol. Included, peer-reviewed articles were published between 2015 to 2020, written in English and did not discuss oral health and clinician training. These healthcare areas were excluded as they represent large, specialized bodies of literature beyond the scope of this review. Online databases (e.g., MEDLINE, CINAHL Plus) were used to locate articles.

**Results:**

Eight thousand six hundred fifty-five potentially relevant articles were identified. Fifty-four were selected for full review. The review yielded 16 relevant articles. Six articles emanated from North America, six from Europe and one each from Africa, Australia, China and India respectively. Most articles used quantitative methods and examined various aspects of healthcare. Studies centered on: indicators; equity policies; evaluating the equitability of healthcare systems; creating and/or testing equity tools; and using patients’ sociodemographic characteristics to examine healthcare system performance.

**Conclusion:**

Although equity is framed as an important component of most healthcare systems’ performance frameworks, the scarcity of relevant articles indicate otherwise. This scarcity may point to challenges systems face when moving from conceptualizing to measuring equity. Additionally, it may indicate the limited attention systems place on effectively incorporating equity into performance frameworks. The disjointed and varied approaches to conceptualizing equity noted in relevant articles make it difficult to conduct comparative analyses of these frameworks. Further, these frameworks’ strong focus on users’ social determinants of health does not offer a robust view of performance. More work is needed to shift these narrow views of equity towards frameworks that analyze healthcare systems and not their users.

## Introduction

Two decades ago, the Institute of Medicine released the seminal report titled ‘Crossing the Quality Chasm: A New Health Care System for the 21^st^ Century’—that identified equity as a key dimension of healthcare quality [1]. Although no universal definition of health equity exists, here, we use Braveman’s inclusive definition of equity as the elimination of avoidable health disparities and its determinants among groups with various levels of privilege based on their socioeconomic status, gender identity, race, education and other categories of difference [2, 3]. Most healthcare systems across the world emphasize the importance of equity in the policy statements, strategies and performance frameworks used to measure the success of these policies and strategies [4]. Here, we define healthcare as the enhancement of one’s health through the diagnosis, prevention or treatment of physical and mental ailments in individuals. We define healthcare systems as an interconnection of multiple individuals, institutions and resources that provide healthcare services that match patients’ needs.

Healthcare systems use various performance frameworks and indicators to monitor their healthcare services and resources as well as identify areas for improvement at the system, community and institutional levels. Public release of this data can impact institutions’ and communities’ reputations and motivate them to enhance their performance [4]. Over the years, scholars such as Braveman [5] have created conceptual frameworks researchers can use as guides to help them determine if healthcare systems are effectively monitoring equity in their systems. Braveman’s multistep, policy-oriented approach included identifying: the groups in question/concern; general issues and information needs relating to equity in health and its determinants; indicators of health status; and major determinants of health status beside healthcare [5].

Although Braveman’s framework was published nearly twenty years ago, it is unclear how it and other frameworks have incorporated equity into healthcare systems’ assessments of their healthcare services and the extent to which scholarship on healthcare system performance has provided guidance on measuring equity as part of performance. As no universal definition of healthcare systems performance exists, here, we define it as indicators, tools and products used to determine how well a healthcare system is performing in terms of, for example, quality of patient care, service costs and care outcomes. For instance, a 2020 review of primarily U.S. literature focused on equity frameworks, potential strategies and measurement considerations in public health practice noted that approaches to measuring health equity differ widely. The limited literature centred on health equity within public health varied in which metrics they suggested to measure health equity [6]. Given the uncertainty and the potential negative implications data gaps can have on patient outcomes, understanding how equity is defined, measured and used to inform healthcare practice is important. As such, we conducted a scoping review to explore how healthcare systems around the world conceptualize equity when considering healthcare system performance.

### Literature search

We used Levac et al.’s approach to conducting scoping reviews [7]. This approach builds upon Arksey and O’Malley’s original scoping review framework. The original framework recommended a five-stage approach: 1) choose a search topic; 2) locate pertinent studies; 3) select relevant studies; 4) chart the collected data; and 5) condense and report the findings of the collected data [7]. Levac et al. [8] extended this approach by including: 6) a consultation with stakeholders to gain additional information sources, views, meaning and applicability to the study; and 7) a discussion about future research, practice and policy implications. Additionally, we sought feedback from a University of Toronto librarian to help develop, assess and revise our scoping review protocol. The authors continuously reviewed the protocol during the search to ensure it would capture potentially relevant articles.

### Search strategy

The search was informed by the question: How is equity conceptualized in healthcare systems when assessing healthcare system performance? To maximize the number of potentially relevant articles, we chose not to select a specific target population, country or health outcome. The literature search was conducted from spring to summer 2021 using six electronic databases: Medline, EMBASE, CINAHL Plus, Sociological Abstracts, Cochrane Library, and PAIS Index; chosen through consultation with the librarian. The key search terms (see Table 1) were modified to maximize search results based on the database being used. Commonly used terms for equity in healthcare system performance and health equity literature were included.

**Table 1:** Examples of search terms used in database searches

Equity	Equality	Disparity	Health
healthcare equity	health inequality	health care disparity	healthcare
health equity	inequality	health status disparity	primary care long term care
health inequity	inequity	health disparity	physiotherapy
equity	equality		mental healthcare
health equity measurement			healthcare performance
health equity policy			healthcare performance reporting
health equity practice			healthcare measurements
health equity frameworks			healthcare performance measurement
			health indicators
			health status
			performance measurement
			determinants of health
			social determinants of health
			social determinants
			public health practice

### Removing duplicates

A total of 10,370 articles were found. After eliminating duplicates from potentially relevant articles (using Zotero reference managing software and through manual elimination), 8655 remained.

### Inclusion and exclusion criteria

The articles were first screened for relevancy based on their titles by two members of the study team. Articles were included if they were: peer reviewed articles; written in English; focused on defining and measuring healthcare equity and equity in primary healthcare systems. The initial search time frame was 2000–2020. We then limited the search to the last five years (2015–2020) to maximize the number of relevant hits and due to the fact that health/(care) equity focused research has become more prevalent in the last five years. The final selected papers were also reviewed by a third team member to ensure they mirrored the inclusion criteria. Articles that did not meet the aforesaid inclusion criteria and those focused on oral health and healthcare provider training were excluded. The last two exclusions were noted as they are beyond the scope of this review and reflect different areas of healthcare and understandings of equity – for example, the role of geographical access to oral health services (Quinonez, C. 2021, oral communication, 5^th^ November) or different aspects of performance (training).

### Article review strategy

The articles’ titles and abstracts were imported into Zotero. Based on the large number of results, two team members split the retrieved articles in half and reviewed their assigned articles. Prior to reviewing their respective articles, the study team sought to ensure review consistency by both assessing the first 100 search results separately. The team then convened to discuss their selection process and reassess the scoping review protocol. This discussion yielded no protocol changes. They then continued their respective reviews and placed the articles’ titles and abstracts into three categories: 1) Include-full review needed; 2) Exclude-full review not needed; and 3) Questionable-full review may be needed.

The team then reconvened to discuss articles placed into the include and questionable categories. Any article disagreements were resolved by discussions with the third team member. Articles selected for full review were downloaded to Zotero. Articles were then divided among two team members for a full screenings. The team met again to discuss their findings.

### Data extraction

Two reviewers extracted data from the full text articles that met the inclusion criteria. Prior to data extraction, 10 articles were randomly selected and the two team members independently extracted information from them. They then met to discuss and review their data extraction process to ensure consistency in their approach. Any disagreements were resolved through discussion. The full text articles were then divided amongst the two team members. This division occurred due to time constraints.

## Results

**Fig. 1:**
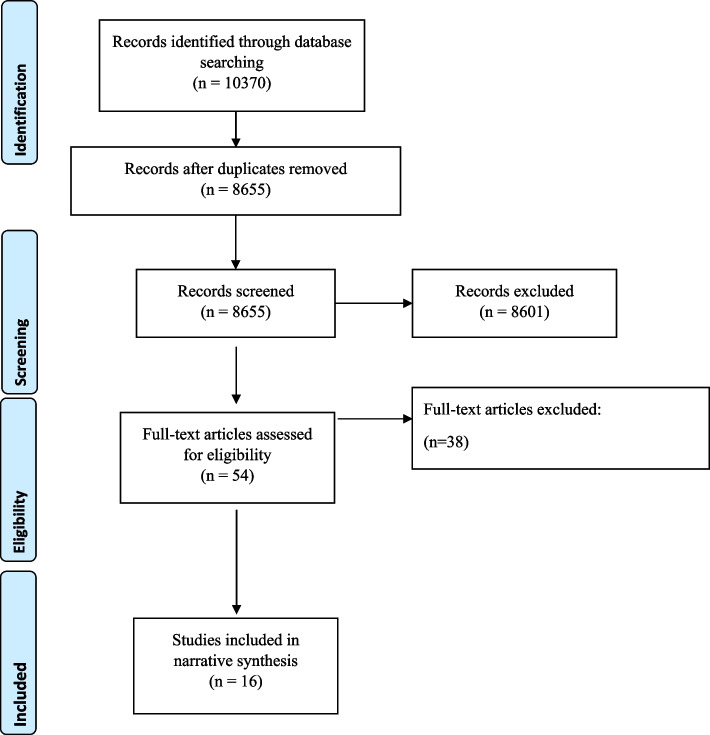
Search Process Showcased in a PRISMA Flowchart for the Scoping Review

Figure 1 shows that application of the inclusion and exclusion criteria led to the exclusion of 8601 articles. A total of 54 articles underwent full text screening. Fully screened articles ultimately excluded from the scoping review identified equity as an important component of healthcare but failed to define and explain equity informed frameworks and tools in their healthcare field under study. Additionally, some excluded studies conflated equity with equality in their exploration of healthcare system performance. This screening yielded 16 articles that met the inclusion criteria (please see Table 1 for an overview of these articles). Definitions of equity in these 16 articles varied widely, ranging from providing no definition [9, 10] to a focus on distributional fairness of healthcare services to populations with differing levels of advantage and disadvantage (e.g., [11–14]). Irrespective of the article’s country of origin or study methodology, these levels often centred on the impact of populations’ socio-economic status on healthcare service access and delivery.

Six studies came from North America (three from Canada and three from the U.S.), another six from Europe (two from the UK, two from Italy and two from Sweden). The remaining studies were based in Sierra Leone (*n* = 1), Australia (*n* = 1), China (*n* = 1) and India (*n* = 1). Half the articles were published in 2018, the rest largely spread out over the other years. Please see Table 2 for an overview of the included articles.

**Table 2:** Summary of included articles

Authors	Year of publication	Country of origin/under study	Study Design	Overview of Data Analysis Tools Used	Indicators Provided
Anderson et al [15]	2018	U.S	Quantitative	n/a -equity roadmap proposal	n/a
Asaria et al [9]	2016	UK	Quantitative	Lower super output area Index of multiple deprivation Slope index of inequity (SII) Relative index of inequality (RII)	Primary care supply Primary care quality Preventable hospitalization Amenable mortality
Buja et al [11]	2015	Italy	Quantitative	unconditional exploratory logistic regression analysis of indicators	Indicators of processes: 1. patients with STEMI-AMI not treated with percutaneous coronary intervention (PCI) 2. older patients with hip fractures undergoing surgery more than 48 h after being admitted to hospital 3. open cholecystectomies 4. women undergoing caesarean section
Burström et al [12]	2017	Sweden	Scoping Review- Mixed Methods	Donabedian's framework for assessing quality of care	n/a
Cohen et al [13]	2018	Ontario	Qualitative	NVivo qualitative data analysis software Content analysis	15 indicators, placed into five categories: 1. Assess/report inequities 2. Modify/orient programs/services 3. Engage in community and multi sectoral collaboration in addressing the health needs of populations through services and programs 4. Lead/support/participate with others to address policies 5. Organizational and development role
Cookson et al [10]	2018	England	Quantitative	Lower super output area Index of multiple deprivation Linear regression Absolute gradient index	Quality of ambulatory care services in managing long term conditions
Freeman et al [16]	2018	Australia	Mixed Methods	QSR NVivo software for thematic analysis	n/a
Lavoie et al [17]	2018	Canada	Mixed Methods	Socio-historical narrative of each clinic Analysis of board meeting minutes and funding contracts for each clinic	n/a
Li et al [18]	2018	China	Quantitative	4-level hierarchical random effects models 4-level regression model	n/a
Lloren et al [19]	2019	United States	Quantitative	hospital‐specific random coefficient for patient dual eligibility status or African American race Sensitivity analyses	n/a
Saia et al [20]	2018	Italy	Quantitative	predictive models multilevel logistic regression	The proportion of patients with heart attacks treated with primary percutaneous coronary intervention (PCI) within 24 h The proportion of in-hospital deaths among patients treated for hearth attacks
Sebastián et al [14]	2017	Sweden	Quantitative	The horizontal inequity index (HII) probit regression the concentration index	Socioeconomic indicator individual disposable income
Shah et al [21]	2015	United States	Quantitative	multivariable analysis descriptive statistics logistic regression models	n/a
Smithman et al [22]	2018	Canada	Quantitative	Social and material area deprivation indexes multiple logistic regression Pearson chi-squared test	n/a
Vallières et al [23]	2016	Sierra Leone	Quantitative	Pearson Chi-Squared test	Maternal health child health sanitation
Ward et al [24]	2020	India	Quantitative	survey-weighted logistic regression survey logistic regression wealth index Benjamini–Hochberg correction	Indicator categories: Reproductive Maternal Newborn Child health Nutrition NOTE: Indicators were relevant to interventions across the continuum of care and health disparities. For each continuum of care domain, researchers also classified the indicators by delivery platform

All included studies noted in general terms, the impact of various social determinants of health on individuals’ overall health as well as healthcare access, quality, treatment and outcomes. In particular, socioeconomic status was frequently identified as a key factor driving variations in healthcare use and outcomes. It is important to note that many studies that reported data on patients’ socioeconomic-status retrieved this information from other data sources such as national censuses as healthcare institutions often did not collect such data from its patients. The potential ramifications of applying national data to regional healthcare institutions were not discussed. Some studies used patients’ socioeconomic status to explore links between socioeconomic disparities and overall healthcare quality (e.g. [9]) and access to healthcare programs [24].

The studies had diverse foci. Studies examined equity in the context of: primary healthcare (*n* = 4) [15–17, 22]; healthcare quality performance (*n* = 1) [9]; healthcare in community settings (*n* = 2) [15–17, 22]; hospital settings (*n* = 4) [10, 11, 19, 20]; primary healthcare reform (*n* = 1) [12]; local public health agencies (*n* = 2) [13, 21]; impact of policy informed changes to medical expenses (*n* = 1) [18]; healthcare access (*n* = 1) [14]; and maternal and child healthcare (*n* = 2) [23, 24].

Studies primarily used quantitative (*n* = 12), followed by mixed-method (*n* = 3), and qualitative (*n* = 1) approaches to collect and analyze study data. Data were frequently taken from pre-existing local or national surveys and/or collected by surveying study participants. The qualitative study used a case study design to help create indicators to monitor and develop health equity and social determinants of health-related actions in a Canadian province’s local public health agencies. Staff members from four agencies were selected to collect indicator data with a standardized worksheet made by the research team and asked to participate in focus groups. Data about the indicators and focus group transcripts underwent content analysis. Mixed method approaches included one-on-one and focus group interviews. Some studies incorporated various indices of deprivation. For instance, a study highlighted the UK National Health Service’s creation of an equity indicator that aimed to provide quality assurance information about the service’s duty to consider decreasing inequalities in healthcare access and outcomes. They used the index of multiple deprivation as one of its measures [10]. Several studies that employed these indices noted they used them as proxies to estimate patients’ socioeconomic status or the socioeconomic status of a particular geographic location. Patients’ level of education was often used as a proxy for socioeconomic status [11, 14, 24]. These estimated statuses were often compared against healthcare access, usefulness and outcomes [9, 11].

### Indicators

Nearly half the articles reviewed used indicators to monitor various aspects of healthcare. Indicators included healthcare performance [9, 20, 24] and hospital process quality [11]. One article used healthcare service delivery, treatment and preventative care as indicators of performance that were then compared across two geographical locations [23]. Conversely, some collected sociodemographic characteristics and others to gain insight into their healthcare services [14, 20, 22]. For instance, one of the aims of a study from an Italian region’s new network for acute myocardial infarction treatment was to determine if the network reduced health inequities among socio-demographic subgroups of populations needing treatment [20]. Two articles used a socioeconomic indicator to help calculate their selected index of inequality [14, 22]. Only one article also noted an additional classification of structural indicators by delivery platform [24].

### Evaluating the equity of healthcare systems

Five studies centered on evaluating the equity of their healthcare systems. Another Swedish study assessed if the principles of horizontal equity were met in terms of healthcare access and explored the impact of various factors on healthcare access inequalities from 2006–2014 in some of the country’s northern counties [14]. Researchers in the U.S. analyzed the degree that local health departments employed strategies and activities to tackle health disparities and health departments’ levels of involvement in activities to counter health disparities in their respective communities [21]. Canadian researchers in Quebec examined the link between social and material deprivation and the chance that individuals had a regular general practitioner (GP) as well as the wait times to get a regular GP through the province’s centralized waiting list [22]. In a Sierra Leone district, researchers identified service delivery gaps of free, essential maternal and child health services for two rural locations [23]. Another study conducted an equity analysis of reproductive, maternal, newborn and child health and nutrition services to determine if the services’ health affects would be the same for the least and most marginalized women in an Indian state [24].

### Equity policies

Three of the 16 articles sought to critically assess the equity impact of particular policies, on their country’s healthcare system performance. This included Burström et al.’s [12] scoping review that assessed evidence on the equity impact of the Swedish government’s Primary Health Care (PHC) Choice Reform. The reform let citizens pick their PHC provider and allowed private PHC providers establish their practices in a region or city of their choosing. The government sought to augment patients’ PHC provider choice, increase the number of privately offered healthcare services and use inter-provider competition to increase healthcare quality and innovation [12]. Another article focused on the impact of a policy that sought to curtail rising drug costs and increase service use in primary health institutions in China [18]. Saia et al. [20] examined the impact of a new organizational model of an Italian region’s healthcare network for heart attacks to help facilitate non-surgical invasive treatment and its effectiveness in decreasing health inequities.

### Creating and/or testing equity tools

Eight studies focused on the creation and/or testing of health equity tools to assess organizations’ commitments to health equity. Although no universal definition of equity tools exist, it is defined here as documents, resources, indicators and measures that seek to assess that state of equity in healthcare systems. Cohen et al. [13] sought to create indicators to monitor and direct health equity and social determinants of health related work in Ontario’s local public health agencies. Preliminary indicators were selected then field tested with the help of various public health agencies and their workers [13]. An article from the U.S. discussed a recently created equity roadmap that aimed to reduce disparities. The roadmap included recommendations about using quality measurements and payments to reduce healthcare disparities and increase health and healthcare equity [15]. A study conducted in Australia developed a framework that assessed regional public healthcare organisations’ contributions to health equity and used it to review the country’s disbanded Australian Medicare Locals [16]. Lavoie et al.’s [17] study discussed the policy affects of a primary healthcare (PHC) intervention that sought to increase capacity for equity oriented healthcare at community health centres that deliver PHC to underserved populations in Canada. Conversely, UK researchers created four summary measures linked to trends in socioeconomic inequity in healthcare access, quality and outcomes that the healthcare system could be held accountable for [9]. A study from an Italian region examined if links existed between social determinants and adherence to four hospital care process quality indicators [11]. The UK study’s equity indicator sought to give quality assurance information about the NHS’ duty to reduce inequalities in healthcare access and outcomes. The study aimed to showcase a new analytical approach that gives healthcare purchasing and planning agencies detailed, current information on the equity aspect of healthcare quality within their patient populations [10]. Finally, U.S. researchers highlighted a new metric that was an extension of pre-existing quality outcomes measures that aimed to highlight within-hospital differences for various patient groups [19].

### Using patients’ sociodemographic characteristics to examine healthcare system performance

As previously noted, many studies collected patients’ or estimated regional sociodemographic data [9, 12, 22]. Data were used to contextualize study findings and frame how various components of the healthcare system performed. Patients’ and/or neighbourhood level socioeconomic statuses were often assessed against healthcare access and distribution of resource data [9, 10, 12, 22]. For instance, Asaria et al. [9] found that neighbourhood reductions in socioeconomic inequalities over a nearly 10 year period yielded significant reductions in inequality in primary care supply and quality. However, inequalities in amenable mortality and preventable hospitalization were mixed. Patient characteristics were also used to contextualize the equity of quality healthcare delivery. Buja et al. [11] compared patient gender, age, citizenship and education to evidence based quality healthcare delivery. Only two studies explicitly included and examined the impact of patients’ race on healthcare disparities. One study included and compared patients’ race to hospital specific disparities in the U.S.. Hospitals with high racial disparities had worse hospital quality [19]. Conversely, another study from the U.S. demonstrated how their equity roadmap could be utilized to reduce disparities in the prevention and control of hypertension in African Americans [15].

## Discussion

To our knowledge, this is the first scoping review that examined the ways health equity is conceptualized when considering healthcare system performance. The significant lack of relevant literature found revealed a disconnect between researchers framing their studies as equity focused and examining their healthcare area of interest through an equity lens. It may also point to a lack of consensus on how equity is defined and used to understand healthcare system performance. More research is needed to better understand this lack of consensus and how to ensure researchers are truly assessing the state of equity in their respective healthcare systems.

This review’s inclusion of studies conducted in high and low-income countries emphasizes healthcare systems’ universal commitment to equity. Although key differences exist in healthcare systems in North America, Europe, Africa and Asia, they provide great insight into the ways these systems attempt to assess the equitability of healthcare quality, performance, access, process, delivery and outcomes. The review’s focus on peer-reviewed articles published in the last five years allowed for a greater understanding of the ways healthcare systems’ current views and measurement of equity are shaping patient care.

All articles acknowledged that social determinants of health affected patients’ health and their outcomes in various healthcare settings. Many sought to examine the impact of these determinants on the equitability of their healthcare system of interest by analyzing patients’ sociodemographic characteristics or using these characteristics as indicators to help review the system’s various parts. However, these examinations and reviews were often rudimentary in nature and merely reaffirmed the impact of these determinants on health without critically examining how equitable the various components of the healthcare systems under review were. This narrow focus on determinants was underscored by numerous articles’ use of national census data to conduct this examination. In some cases, patients’ socio-economic statuses were framed as being so important to studying equity in healthcare systems that researchers used various indices as proxies to estimate patients’ or geographical areas’ statuses when this data was unavailable. The use of these indices point to a lack of routine patient socio-demographic data collection leading many systems to rely on other, more general data sources to gain insight into their systems. As such, study findings may not provide an accurate picture of their patients’ social determinants of health and the state of equity in these systems.

Numerous studies used sociodemographic characteristics such as gender and age to identify discrepancies in patients’ access to care and other healthcare gaps without critically discussing how equity was defined and chosen measurement tools used to help capture healthcare system performance through an equity lens. These characteristics were viewed through a cis- and heteronormative lens, erasing the voices of individuals with various sexual orientations and gender identities. As the studies predominantly focused on adult patients, there is silence on whether or not differences exist in health equity performance measures for those under 18 years of age. Additionally, only two articles explicitly reviewed the impact of race on health disparities. As racism is known the negatively impact healthcare quality, health outcomes [25] and provision [26], more research should be conducted to see how or if the impact of racism is being captured when assessing healthcare system performance. Additional work is needed to move beyond this basic, patient-focused conceptualization of healthcare system performance.

Most studies were quantitative and used common statistical approaches to analyzing the study data. Employing qualitative or more mixed method research designs may have further illuminated the nuanced underpinnings of the ways equity is viewed, conceptualized and then used in healthcare settings to inform system planning.

Some articles critically examined the equity impact of particular equity policies of their country’s healthcare system performance and quality. Researchers noted that inequities existed in their systems and sought to determine if policy informed strategies created to address them worked as intended. While these strategies often led to improvement in differences in equity, disparities in patients’ health status and/or the social determinants of health often remained. This juxtaposition of findings reconfirms the need for additional resources to be diverted to addressing many of the social determinants of health that healthcare systems may have the capacity to address. Additionally, these articles may point to the beginning of a shift from identifying health inequities to actively working towards addressing them. As noted by half the included articles, some researchers have developed equity tools to monitor equity practices and system performance in various healthcare settings. These tools allow for a greater understanding of how systems are truly supporting patients’ health and which measures are actually capturing the state of equity in healthcare system performance.

## Limitations

Although this scoping review used a rigorous and thorough search strategy, limitations exist. Only peer-reviewed articles written in English were included in the review. This may have prevented the location of other relevant, non-English articles. A future review of grey literature may help reveal promising areas of health equity research. Every effort was made to identify the various terms used to discuss equity in the literature to locate relevant articles. However, it is possible that some relevant articles that did not include these common terms were excluded. Our selected search timeline of 2015–2020 could have eliminated other pertinent articles from being identified. However, as discussions of equity and healthcare have come to the forefront in recent years, it was important to gain insight on the current ways this discourse was being taken up in the literature.

Variations in the types of sociodemographic data collected from patient focused studies were noted. This makes it difficult to discern what components of this data may be vital aspects to consider when reviewing equity informed healthcare system performance. Most studies did not critically discuss the impact of patient age, race and gender identity on their findings. As these intersecting identities are known to impact individuals’ access to appropriate, high quality care, future studies should address this literature gap. Some researchers noted that the health administrative data used in their studies did not include patients’ socioeconomic data which prompted them to use neighbourhood census data as a proxy (e.g., [12, 13]). This use may not truly reflect the diversity of the population under study as some individuals may have a high socio-economic status but live in low socio-economic status neighbourhoods. Finally, the indices used to analyze data in many of the studies ultimately focused on tabulating patients’ socioeconomic status in the context of healthcare. Researchers’ dependence on these indices may have prevented the use of other analytical tools that would better capture the level of equitable healthcare quality, processes and performance.

## Conclusion

This scoping review provided an international overview of the ways equity is being conceptualized when assessing healthcare system performance. Although equity is considered a cornerstone of healthcare in most countries, findings indicate that the analysis of equity within healthcare systems is often disjointed, varied and centered on reporting patient outcomes. Patients’ and geographic areas’ sociodemographic characteristics continue to be used to assess the state of equity in these systems. Although these characteristics can help inform healthcare planning and the types of services offered, they do not provide robust insight into how healthcare systems are performing in an equitable fashion. It is important that institutions using equity lenses in their work seek out and incorporate equity frameworks and tools when conducting healthcare performance assessments. Failure to do so will merely reinforce a longstanding cycle where patients, and not healthcare systems are the ones under scrutiny.

## Data Availability

Not applicable. No datasets were created or analyzed during this study.
